# The evolutionary origin of the collective unconscious and the appeal of research on Neanderthals and other ancient humans

**DOI:** 10.3389/fpsyg.2026.1857884

**Published:** 2026-07-16

**Authors:** Marcelo R. S. Briones

**Affiliations:** Center for Medical Bioinformatics, Escola Paulista de Medicina, Federal University of São Paulo (UNIFESP), São Paulo, Brazil

**Keywords:** ancient genomics, archetypes, collective unconscious, evolutionary psychology, Homo heidelbergensis, human evolution, Neanderthals, science communication

## Abstract

Why does research on Neanderthals attract public attention far beyond its immediate scientific relevance? Such fascination may reflect more than intellectual curiosity; it may involve the activation of deep symbolic structures that Carl Jung termed the collective unconscious. Integrating evolutionary biology, genomics, paleoanthropology, and psychology, I address a question Jung did not explicitly pose: when, along the human evolution, did the collective unconscious originate? I argue that this structure emerged gradually rather than suddenly. *Homo erectus* established a cognitive floor characterized by basic schemas of fear, group cohesion, and hierarchy, without evidence of symbolic elaboration. *Homo heidelbergensis*, the common ancestor of Neanderthals and *Homo sapiens*, is proposed as the strongest candidate for the emergence of proto-archetypal cognition, given its enlarged brain, social complexity, and possible forms of mortuary behavior. The symbolic system was operational in Neanderthals and archaic *H. sapiens* and became fully visible with the symbolic expression of the Upper Paleolithic. I further introduce the concept of the “incorporated other” to explain the distinctive psychological salience of Neanderthals and Denisovans. Unlike other extinct organisms, these archaic humans remain present in contemporary populations through genetic introgression, collapsing boundaries between self and other, past and present. I propose that this condition contributes to public fascination in ways not fully explained by resemblance, category ambiguity, intuitive essentialism, or the uncanny. The “incorporated other” predicts that genetic incorporation itself increases perceived self-relevance and engagement, even when similarity is held constant. Neanderthals are therefore psychologically salient because they are simultaneously extinct and biologically present.

## Introduction

Research on Neanderthals consistently attracts public attention that exceeds its immediate scientific or biomedical relevance. Studies on ancient genomes, cognition, human origins, and extinct pathogens routinely generate global media coverage and intense non-specialist engagement (Green et al., 2010; Vernot and Akey, 2014; Hoffmann et al., 2018). This response persists even when findings have little practical or clinical significance.

Why do Neanderthals matter so much to the public? Standard explanations point to curiosity about origins, technological novelty, or possible medical applications. These factors contribute, but they do not fully account for the scale and persistence of public fascination. Many highly publicized studies offer no clear translational benefit yet still provoke strong emotional and cultural reactions.

Recent work on ancient pathogens illustrates this pattern clearly. Reports that early humans carried oncogenic or persistent viruses have attracted extraordinary attention, despite limited immediate medical implications (Agoni et al., 2012; Guellil et al., 2022; de-Dios et al., 2023; Ferreira et al., 2024; Yazigi et al., 2026). An article by Bridget Alex in *Science* on ancient human papillomavirus, discussing our work on paleovirology, exemplifies this phenomenon, linking contemporary disease to deep evolutionary history and generating widespread public interest (Ferreira et al., 2024; Alex, 2025; Yazigi et al., 2026). Comparable public reactions have followed major discoveries involving Neanderthal genomes, Denisovan ancestry, cave art, and the sequencing of archaic human remains, suggesting that the phenomenon extends beyond any particular research program or pathogen-related finding (Green et al., 2010; Reich et al., 2010).

Here I argue that Neanderthals occupy a distinctive psychological and symbolic position as an “incorporated other”: an extinct human lineage that is nevertheless genetically present in many contemporary populations (Green et al., 2010; Vernot and Akey, 2014). This unusual status collapses intuitive boundaries between self and other, past and present, generating a level of cognitive and emotional salience that few other extinct organisms can achieve. I further propose that this fascination may be understood, in part, through the lens of Jungian analytical psychology, specifically, the concept of the collective unconscious, and examine the evolutionary origins of such deep symbolic structures.

Importantly, this position is shared, in part, by archaic anatomically modern humans (AMHs) who overlapped with Neanderthals in time and space. These early *H. sapiens* populations function as historical witnesses to Neanderthal existence, interaction, and admixture, reinforcing continuity across deep evolutionary time.

## An unstable evolutionary category

For more than a century, Neanderthals have functioned as an unstable symbolic category. Early scientific and popular representations portrayed them as brutish, cognitively inferior, and evolutionarily “failed” (Stringer and Gamble, 1993; Trinkaus and Shipman, 1993). These depictions reflected social and cultural assumptions as much as empirical evidence.

Over time, archaeological and genetic research challenged this image. Evidence for complex tool use, social organization, symbolic practices, and interbreeding with modern humans undermined simple narratives of inferiority (Hoffmann et al., 2018). Neanderthals increasingly appeared less as evolutionary dead ends and more as close relatives with sophisticated behavior.

This gradual revision did not stabilize their symbolic status. Instead, it intensified ambiguity. Neanderthals came to be seen as both similar to and different from modern humans, both familiar and alien. Such ambiguity is psychologically powerful. Categories that resist clear classification tend to attract sustained attention and emotional investment.

Archaic AMHs further complicate this picture. Individuals such as those represented by early Eurasian *H. sapiens* populations shared landscapes, resources, and social environments with Neanderthals. They were contemporaries, neighbors, and sometimes biological partners. Their presence transforms Neanderthals from distant ancestors into participants in a shared human world.

## Genetic incorporation and symbolic proximity

The discovery of widespread Neanderthal introgression in non-African populations transformed public understanding of human evolution (Green et al., 2010). Neanderthals were no longer merely distant ancestors or evolutionary side branches. They became biological contributors to living humans, an incorporated other.

This shift has important psychological consequences. Clear distinctions between “us” and “them” support processes of projection and moral distancing. When boundaries blur, identification increases.

Genetic incorporation reduces categorical distance. Neanderthals are no longer entirely external. They are, in a literal sense, part of “us.” This makes it more difficult to treat them as safely distant figures onto which undesirable traits can be projected.

Few extinct organisms occupy this position. Dinosaurs, mammoths, and early hominins remain clearly separated from modern identity. Neanderthals do not. Their genetic presence anchors evolutionary history in everyday biology, transforming abstract ancestry into embodied reality.

The concept of the incorporated other requires distinction from several adjacent concepts in psychology and anthropology. Freud's uncanny (Unheimlich) emerges when something simultaneously familiar and unfamiliar destabilizes ordinary perception (Freud, 1919). Kristeva's abject concerns the disruption of boundaries between self and other through exclusion, contamination, or bodily ambiguity (Kristeva, 1982). Anthropological theories of liminality similarly emphasize entities that occupy unstable classificatory positions (Turner et al., 1969).

The incorporated other differs from each of these. It refers to an entity that is simultaneously external and constitutive: an extinct lineage that remains biologically present within the populations that contemplate it. Neanderthals are neither wholly self nor wholly other. Their genomes persist within living humans, yet they remain historically distinct and evolutionarily separate. This combination of biological incorporation, symbolic ambiguity, and historical absence creates a psychological configuration not fully captured by existing concepts.

The concept of the incorporated other is intended at three interconnected levels. Ontologically, Neanderthals are incorporated through documented genetic introgression. Psychologically, this condition may increase their salience as objects of identification and projection. Communicatively, scientific narratives repeatedly emphasize this relationship, reinforcing their distinctive position within public imagination (Vernot and Akey, 2014).

The Denisovans deepen this picture considerably. Identified initially from a finger bone and teeth recovered from Denisova Cave in the Altai Mountains of Siberia, this sister lineage to the Neanderthals contributed approximately 4–6% of its genetic material to the genomes of present-day Melanesians, with evidence of distinct introgression events reaching populations across Southeast Asia, Oceania, and as far as the Philippines (Reich et al., 2010; Ongaro and Huerta-Sanchez, 2024). Recent genomic analyses have revealed at least three separate introgression events from distinct Denisovan populations, each showing different levels of relatedness to the sequenced Altai individual, indicating a complex and geographically extensive archaic presence across Asia during the Late Pleistocene.

Like Neanderthal introgression, some of these Denisovan-derived sequences appear to have conferred adaptive advantages, including immune function and high-altitude physiology. The Denisovans are in one important respect, even more symbolically striking than Neanderthals: their fossil record consists of only fragmentary remains, yet their biological imprint on living populations is unmistakable. They are, in a sense, the most purely genomic form of the incorporated other: present in living bodies but largely absent from the archaeological data. This asymmetry between biological persistence and morphological absence may itself be a novel dimension of the “incorporated other” phenomenon, raising the question of whether populations can carry deep evolutionary entanglement with an ancestor whose appearance and cognitive life remain almost entirely unknown.

## The collective unconscious and its evolutionary origins

### Jung and the Neanderthals: when does the collective unconscious begin?

For Carl Gustav Jung, the collective unconscious does not “begin” at a historical moment. It is structural, it arises with the human species itself. The collective unconscious is a set of universal forms (archetypes) that do not depend on any specific culture. Therefore, by Jungian logic, they emerge together with the modern human mind (Jung, 1959).

However, the question of Neanderthals makes this picture more complex and considerably more interesting. Neanderthals possessed deliberate burial practices, possible symbolism, and use of pigments (Hoffmann et al., 2018). This suggests a rudimentary symbolic life. In Jungian terms, they likely operated with proto-archetypes: representations of death, the mother, the group, danger. Whether they achieved the same level of symbolic elaboration as *H. sapiens* remains an open question.

It has been proposed that the clearest expression of what is recognized as archetypal imagery appears with the Upper Paleolithic, approximately 40,000 years ago: cave art, hybrid human-animal figures, and more complex ritual structures (d'Errico and Stringer, 2011). Here one can identify explicit archetypes like the shadow (in the form of dangerous animals), the Self or totality (hybrid figures), and spirit or transcendence (ritual contexts) (Conard, 2009). This expression should not be confused with the origin of the underlying structures because the symbolic expression of the Upper Paleolithic makes the collective unconscious visible but not that it necessarily started at this point.

### Alternative cognitive frameworks and the question of human fascination

The interpretation proposed here is not the only possible explanation for the remarkable public fascination with Neanderthals and other archaic humans. Several contemporary frameworks from cognitive science, evolutionary psychology, anthropology, and science communication offer complementary accounts.

One possibility derives from research on intuitive essentialism, which suggests that humans naturally assume that biological categories possess hidden underlying essences (Gelman and Hirschfeld, 1999; Gelman, 2003). From this perspective, questions concerning human origins, ancestry, and extinct relatives may attract attention because they bear directly on intuitions about what humans fundamentally are.

A second explanation emerges from work on liminality, category violation, and boundary figures (Turner et al., 1969; Boyer, 2001; Douglas, 2003). Neanderthals occupy an unstable conceptual position: neither fully human nor fully non-human, neither ancestor nor stranger. Such ambiguous categories have long been recognized as psychologically salient and culturally productive. Related work in the cognitive science of religion has similarly argued that minimally counterintuitive and category-violating concepts attract disproportionate attention and memorability (Boyer, 2001).

Research on mind perception and the uncanny provides a third perspective (Freud, 1919). Human-like beings that resemble us closely, while remaining recognizably different, often evoke disproportionate emotional responses. Neanderthals may occupy a unique position along this continuum.

The framework developed here does not seek to replace these explanations. Rather, it proposes that Jung's notion of the collective unconscious may be reinterpreted as a higher-level symbolic description of why such cognitive predispositions repeatedly organize themselves around recurring themes of ancestry, identity, mortality, kinship, and otherness. In this view, archetypes are not inherited symbolic images, but recurrent patterns of meaning generated by a shared evolutionary architecture.

Contemporary evolutionary and cognitive psychology generally explains these phenomena through evolved cognitive mechanisms, core knowledge systems, intuitive essentialism, agency attribution, mind perception, and related domain-specific processes rather than through Jung's original formulation of the collective unconscious (Gelman and Hirschfeld, 1999; Boyer, 2001). The Jungian framework employed here is therefore intended as a complementary interpretive vocabulary rather than as a competing empirical theory.

The incorporated other framework also generates predictions that differ, in principle, from similarity-based or uncanny accounts. If genetic incorporation contributes independently to psychological salience, individuals should show greater engagement with an extinct hominin described as contributing DNA to their own ancestry than with an equally human-like extinct lineage lacking such incorporation. Under similarity-based models, both lineages should elicit comparable responses when morphological resemblance is held constant. The incorporated other framework predicts greater perceived self-relevance, curiosity, and emotional investment in the genome-present condition. Denisovans may provide a natural test case. Despite their limited visual and phenotypic presence, public interest often focuses on hidden ancestry, invisible inheritance, and biological continuity. To the extent that such fascination persists despite weak resemblance-based cues, it would support the hypothesis that incorporation contributes explanatory value beyond similarity alone.

### Placing the origin in the evolutionary tree

Throughout this discussion, the collective unconscious is used in a heuristic sense, referring not to inherited symbolic contents nor to a directly observable biological structure, but to species-typical dispositions that recurrently organize symbolic interpretation and emotional salience.

**Figure 1:**
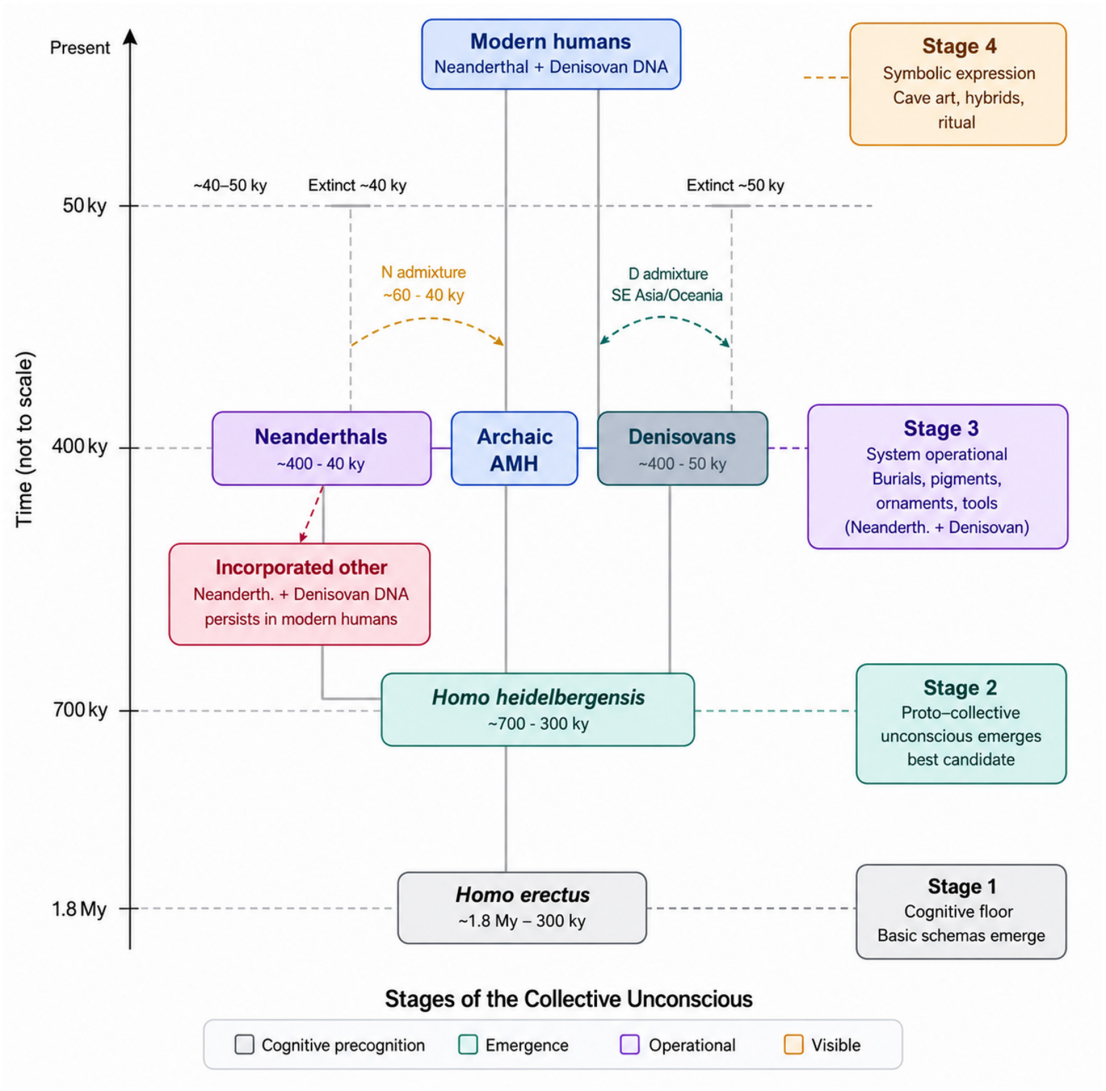
Four evolutionary stages along the human phylogenetic tree, color-coded by the proposed role in the emergence of the collective unconscious: Gray—Stage 1 (*H. erectus*): the cognitive floor. Tool use, fire, and social care provide the basic universal schemas (fear, group, hierarchy), but no evidence yet of symbolic elaboration. Green—Stage 2 (*H. heidelbergensis*): the best candidate for the proto–collective unconscious. Common ancestor of both Neanderthals and *H. sapiens*, with a larger brain, complex cooperation, and possible early funerary practice. Purple—Stage 3 (Neanderthals + Denisovans + archaic AMH): the system is operational. Burials, pigments, and ornaments attest to proto-archetypes in action; archaic AMHs function as evolutionary witnesses. Amber—Stage 4 (Upper Paleolithic): the symbolic expression (~40–50 ky) such as cave art, hybrid figures and rituals. This is when the collective unconscious becomes archaeologically visible, not when it began. The dashed admixture arc shows Neanderthal and Denisovan introgressions into modern humans (~60–40 ky), and the coral box captures the “incorporated other” argument. My = million years and ky = thousand years.

The evolutionary stages proposed below should therefore be understood as a theoretical model rather than a claim of direct empirical identification. Although a single moment of origin cannot be pinpointed, a plausible evolutionary range can be proposed. Four key zones merit consideration (Figure 1):

- ***H. erectus* (~1.8 million−300,000 years ago):** The first evidence of more organized behavior, consistent tool use, controlled fire, and possible social care (Roebroeks and Villa, 2011). There is sufficient cognitive complexity for basic universal schemas like fear, group cohesion, hierarchy, but no strong evidence yet for elaborate symbolism. This represents a cognitive floor, not an emergence zone.
- ***H. heidelbergensis* (~700,000−300,000 years ago):** The common ancestor of both *H. sapiens* and Neanderthals. Signs of complex cooperation and long-term planning are evident in the archaeological record. Possible intentional deposition of the dead and forms of mortuary behavior have been proposed, although these interpretations remain debated (Arsuaga et al., 1997; Stringer, 2012). This is the strongest candidate for a “proto–collective unconscious”: a larger brain, more complex social life, and the beginnings of symbolic representation. If one accepts the heuristic framework proposed here, *H. heidelbergensis* represents the most plausible evolutionary locus for the emergence of proto-archetypal cognition. This should not be interpreted as a claim of direct empirical demonstration but rather as a theoretical inference drawn from converging archaeological, anatomical, and behavioral evidence. The distinction proposed here between the cognitive floor represented by *H. erectus* and the proto-archetypal threshold represented by *H. heidelbergensis* is necessarily heuristic but can be operationalized. *H. erectus* exhibits evidence of planning, technological persistence, fire use, and social cooperation, all of which are compatible with species-typical cognitive schemas. By contrast, the proto-archetypal stage proposed for *H. heidelbergensis* would require evidence of symbolic representation, social abstraction extending beyond immediate practical needs, intentional treatment of the dead, long-term cooperative planning involving group identity, or behaviors suggesting the attribution of shared meaning to events or places. Archaeological signatures that could support this distinction include recurrent mortuary practices, deliberate symbolic marking of objects or locations, and socially transmitted behaviors whose function extends beyond direct survival. Conversely, the absence of such evidence would weaken the proposed proto-archetypal threshold.
- **Denisovans (400,000−50,000 years ago):** Denisovans came from a lineage derived from *H. heidelbergensis*, which in turn descends from *H. erectus* (Reich et al., 2010). However, this was not a simple, linear process. *H. erectus* left Africa (~2 million years ago). A derived population gave rise to *H. heidelbergensis* (~600–700 thousand years ago). From *H. heidelbergensis* in Europe evolved into Neanderthals and in Asia, Denisovans. However, Denisovans show genetic admixture with an even older erectus-like lineage, therefore not just “Asian heidelbergensis,” they are a mosaic population. Neanderthal–Denisovan split: ~400–450 thousand years ago.
- **Neanderthals** **+** ***H. sapiens* (~400,000 years ago onward):** Burials, pigments, and ornaments are clearly attested; art is unambiguous in *H. sapiens* and debated in Neanderthals. The symbolic system is by this stage operational, producing recognizable archetypal symbols in action.
- **The Upper Paleolithic florescence of symbolic expression (~50,000–40,000 years ago):** Cave art, implicit mythology, hybrid figures. This is not the beginning of the collective unconscious, but the moment when it becomes fully and unambiguously visible in the archaeological record (Conard, 2009; d'Errico and Stringer, 2011).

In summary: the cognitive base appears in *H. erectus*. The likely emergence of proto-archetypal structures occurs in *H. heidelbergensis* and the full functioning is evident in both Neanderthals and *H. sapiens* (Arsuaga et al., 1997). The clear, datable expression arrives with the Upper Paleolithic. Therefore, it can be hypothesized that the evolutionary precursors of what Jung later described as the collective unconscious became established in *H. heidelbergensis* (Figure 1).

This evolutionary framing is not merely speculative. It aligns with current neurobiological accounts of the social and emotional brain, the deep conservation of limbic and subcortical circuits across mammals, and the growing evidence that symbolic cognition required not one but a series of incremental biological and cultural thresholds. It also raises a question that may eventually be addressable through epigenetic and comparative genomic research: what neural or epigenetic architecture might sustain these universal patterns across generations and across closely related species?

Evidence from African Middle Stone Age sites such as Blombos Cave suggests that symbolic behaviors, including pigment use and engraved objects, were present well before the European Upper Paleolithic (Henshilwood et al., 2002). Accordingly, the Upper Paleolithic may be understood not as the origin of symbolism but as a particularly visible florescence of symbolic expression.

### Historical intuitions about phylogenetic depth

Long before the advent of ancient genomics, some psychologists speculated that human subjectivity might reflect deep evolutionary conditions. Carl Jung proposed that symbolic structures could have phylogenetic depth, shaped by recurrent species-level experience (Jung, 1959). Similarly, Sándor Ferenczi suggested that aspects of human psychology might retain traces of earlier evolutionary environments (Ferenczi, 1924).

These ideas were necessarily conjectural and lacked empirical support. They did not imply the transmission of specific memories or symbols but reflected an intuition that biological history might shape psychological organization in enduring ways.

Although such theories are not supported as literal mechanisms, the discovery of Neanderthal admixture gives new empirical context to their underlying question: to what extent does evolutionary history remain biologically incorporated in living humans? What earlier theorists approached metaphorically is now observable at the molecular level. The evolutionary archaeology of the collective unconscious sketched above provides one framework within which this question can be explored with fresh rigor.

## Biological continuity and cultural interpretation

The salience of Neanderthals can be understood within multi-level perspectives on inheritance and culture that distinguish genetic, epigenetic, behavioral, and symbolic dimensions of evolution (Jablonka et al., 2014). Symbolic systems are transmitted socially rather than biologically yet are shaped by biological relationships and constraints.

Neanderthals occupy a rare position at the interface of these levels. Genetic continuity establishes material relatedness, while cultural reconstruction depends on modern scientific and media narratives. This does not imply that symbolic content is biologically inherited. Rather, biological relatedness modulates how symbolic information is perceived and valued. Information about close relatives is more emotionally salient than information about distant ones. Neanderthals are unusually close and unusually absent.

Ancient pathogens amplify this effect. Viruses, such as HPV16, herpesviruses, and adenoviruses function as biological witnesses of contact, continuity, and vulnerability. They link hominin interaction to contemporary disease landscapes, further collapsing temporal distance.

## Intergenerational effects and background dispositions

Research on transgenerational epigenetic effects has demonstrated that severe environmental experiences can influence physiological and behavioral dispositions across generations (Meaney and Szyf, 2005; Franklin et al., 2010; Yehuda et al., 2016). Such findings concern inherited sensitivities and affective tendencies rather than the transmission of symbolic representations. This discussion of epigenetics is intended only as an analogy and not as a proposed mechanism for the inheritance of archetypes, symbolic contents or collective memories.

These background dispositions shape how people process uncertainty, risk, and identity-related information (Dias and Ressler, 2014). Although speculative in this context, it is plausible that inherited affective tendencies contribute to the intensity with which evolutionary narratives are received.

In this restricted sense, biological inheritance may influence not what people think about Neanderthals, but how strongly they respond to such information. The Jungian notion of the collective unconscious, understood not as a mystical inheritance but as an evolved architecture of deep affective and symbolic dispositions, offers a conceptual vocabulary for this kind of effect that is worth revisiting in light of current epigenetic and neuroscientific evidence.

## Extinction and evolutionary contingency

Neanderthals confront modern audiences with the fact that cognitive sophistication does not guarantee survival. Despite technological skill and social complexity, they disappeared (Stringer and Gamble, 1993). This challenges linear narratives of progress and human exceptionalism. It highlights the contingency of evolutionary success.

From a psychological perspective, Neanderthals function as a salient counterfactual: a closely related human form that failed to persist. Pathogen-based hypotheses of extinction, whether ultimately supported or not, reinforce this message by foregrounding shared biological vulnerability. They embody the possibility that “we” might not have survived.

In Jungian terms, this activation of contingency and vulnerability may itself touch on archetypal territory, what Jungian psychology metaphorically describes as the shadow of mortality, and the fragility of what we take to be the human. The emotional charge that attaches to Neanderthal extinction may not be merely intellectual empathy but something structurally deeper: an encounter, mediated through science, with one of the oldest affective patterns in our evolved mental architecture.

## Implications for science communication

Public engagement with ancient human research reflects concerns about ancestry, hybridity, identity, vulnerability, and loss, not merely informational deficits. Communication strategies that ignore these dimensions risk misinterpreting audience responses.

Recognizing the symbolic status of Neanderthals encourages communicators to address the existential implications of evolutionary research rather than treating them as peripheral. This is particularly important in contexts involving ancient pathogens, where fear, fascination, and identity concerns intersect.

The framework proposed here, integrating evolutionary biology, Jungian analytical psychology, and paleoanthropology, may offer a more complete account of why findings about Neanderthals and archaic humans routinely escape the boundaries of specialist discourse and enter broader cultural conversation.

## Limitations

This perspective necessarily involves substantial inferential uncertainty. The symbolic life of extinct hominins cannot be observed directly and must be reconstructed from fragmentary archaeological, anatomical, and genetic evidence. Consequently, any attempt to locate the emergence of proto-archetypal cognition within a specific evolutionary lineage should be understood as a heuristic proposal rather than a definitive historical reconstruction.

The present argument also does not imply that Jungian archetypes constitute directly identifiable biological entities. Rather, Jungian terminology is employed as an interpretive framework through which recurring symbolic patterns may be related to species-wide cognitive and emotional dispositions shaped by evolutionary history.

Denisovans may represent a limiting case for the incorporated-other framework. Unlike Neanderthals, they possess little visual or cultural presence in the public imagination. Their salience may therefore derive less from identification and projection than from the paradox of an ancestor who is genetically present yet phenotypically absent. If Neanderthals are incorporated others with a face, Denisovans may be incorporated others without one.

Finally, the concept of the “incorporated other” remains a theoretical proposal requiring empirical examination. Future research in science communication, cultural psychology, and experimental psychology could test whether genetic incorporation, perceived relatedness, and symbolic ambiguity independently contribute to public engagement with archaic human research.

## Conclusion

Neanderthals continue to captivate public imagination because they occupy a unique position as an incorporated other: genetically continuous yet historically extinct (Green et al., 2010). Together with archaic AMHs and ancient pathogens, they collapse boundaries between self and other, past and present, stability and contingency. They are simultaneously ancestors, relatives, witnesses, and losses. As empirical knowledge has increased, this configuration has become more pronounced rather than less. Precision has intensified relevance and data have deepened meaning.

The integration of Jungian theory into this account is not an appeal to mysticism but a recognition that evolutionary biology and depth psychology may be asking the same question from different directions: how deeply does our evolutionary past remain structurally present in who we are? The evidence from ancient genomics, epigenetics, and paleoarchaeology suggests that the answer is: more deeply, and more verifiably, than was previously imaginable.

Unlike Freud's uncanny, which emerges from the return of the familiar within the unfamiliar, or Kristeva's abject, which concerns the destabilization of boundaries through exclusion and contamination, the incorporated other is simultaneously external and constitutive (Freud, 1919; Kristeva, 1982). It denotes an extinct lineage that remains biologically present within the populations that contemplate it. The concept therefore combines evolutionary relatedness, symbolic ambiguity, and genetic incorporation in a configuration not fully captured by existing psychological or anthropological categories.

If the collective unconscious as a structure emerges, as I have argued, in the lineage of *H. heidelbergensis*, then Neanderthals are not merely objects of our curiosity (Figure 1). They are, in a meaningful sense, likely co-inheritors of the same deep symbolic architecture that still operates in every human mind that encounters them.

Neanderthals matter not only because of what they reveal about the past, but because of what they implicitly say about who we are, how fragile we are, and how deeply our present remains entangled with deep evolutionary time.

## Data Availability

The original contributions presented in the study are included in the article/supplementary material, further inquiries can be directed to the corresponding author.

## References

[B1] Agoni L. Golden A. Guha C. Lenz J. (2012). Neandertal and Denisovan retroviruses. Curr. Biol. 22, R437–R438. doi: 10.1016/j.cub.2012.04.049 22677281

[B2] Alex B. (2025). Two ancient humans, including famed “Iceman,” had cancer-causing virus. Science, 2025 December, 23.

[B3] Arsuaga J. L. Martinez I. Gracia A. Lorenzo C. (1997). The Sima de los Huesos crania (Sierra de Atapuerca, Spain). a comparative study. J. Hum. Evol. 33, 219–281. doi: 10.1006/jhev.1997.0133 9300343

[B4] Boyer P. (2001). Religion Explained: The Evolutionary Origins of Religious Thought. New York, NY: Basic Books.

[B5] Conard N. J. (2009). A female figurine from the basal Aurignacian of Hohle Fels Cave in southwestern Germany. Nature 459, 248–252. doi: 10.1038/nature07995 19444215

[B6] de-Dios T. Scheib C. L. Houldcroft C. J. (2023). An adagio for viruses, played out on ancient DNA. Genome Biol. Evol. 15:evad047. doi: 10.1093/gbe/evad047 36930529 PMC10063219

[B7] d'Errico F. Stringer C. B. (2011). Evolution, revolution or saltation scenario for the emergence of modern cultures? Philos. Trans. R. Soc. Lond. B Biol. Sci. 366, 1060–1069. doi: 10.1098/rstb.2010.0340 21357228 PMC3049097

[B8] Dias B. G. Ressler K. J. (2014). Parental olfactory experience influences behavior and neural structure in subsequent generations. Nat. Neurosci. 17, 89–96. doi: 10.1038/nn.3594 24292232 PMC3923835

[B9] Douglas M. (2003). Purity and Danger: An Analysis of Concepts of Pollution and Taboo. London: Routledge. doi: 10.4324/9780203361832

[B10] Ferenczi S. (1924). Thalassa A Theory of Genitality. Available online at: http://archive.org/details/in.ernet.dli.2015.112556 (Accessed April 16, 2026).

[B11] Ferreira R. C. Alves G. V. Ramon M. Antoneli F. Briones M. R. S. (2024). Reconstructing prehistoric viral genomes from neanderthal sequencing data. Viruses 16:856. doi: 10.3390/v16060856 38932149 PMC11209150

[B12] Franklin T. B. Russig H. Weiss I. C. Gräff J. Linder N. Michalon A. . (2010). Epigenetic transmission of the impact of early stress across generations. Biol. Psych. 68, 408–415. doi: 10.1016/j.biopsych.2010.05.036 20673872

[B13] Freud S. (1919). The “Uncanny”. in The Standard Edition of the Complete Psychological Works of Sigmund Freud, ed. J. Strachey (Ed. and Trans.), Vol. 17 (London: Hogarth Press), 217–256. Available at: https://docslib.org/doc/2804589/freud-s-1919-the-uncanny-the-standard-edition-of-the-complete (Accessed May 31, 2026).

[B14] Gelman S. A. (2003). The Essential Child: Origins of Essentialism in Everyday Thought. Oxford: Oxford University Press. doi: 10.1093/acprof:oso/9780195154061.001.0001

[B15] Gelman S. A. Hirschfeld L. A. (1999). “How biological is essentialism?” in Folkbiology, eds. D. Medin, and S. Atran (Cambridge, MA: MIT Press), 403–446. doi: 10.7551/mitpress/3042.003.0013

[B16] Green R. E. Krause J. Briggs A. W. Maricic T. Stenzel U. Kircher M. . (2010). A draft sequence of the neandertal genome. Science 328, 710–722. doi: 10.1126/science.1188021 20448178 PMC5100745

[B17] Guellil M. van Dorp L. Inskip S. A. Dittmar J. M. Saag L. Tambets K. . (2022). Ancient herpes simplex 1 genomes reveal recent viral structure in Eurasia. Sci. Adv. 8:eabo4435. doi: 10.1126/sciadv.abo4435 35895820 PMC9328674

[B18] Henshilwood C. S. d'Errico F. Yates R. Jacobs Z. Tribolo C. Duller G. A. T. . (2002). Emergence of modern human behavior: middle stone age engravings from South Africa. Science 295, 1278–1280. doi: 10.1126/science.1067575 11786608

[B19] Hoffmann D. L. Angelucci D. E. Villaverde V. Zapata J. Zilhão J. (2018). Symbolic use of marine shells and mineral pigments by Iberian Neandertals 115,000 years ago. Sci. Adv. 4:eaar5255. doi: 10.1126/sciadv.aar5255 29507889 PMC5833998

[B20] Jablonka E. Lamb M. J. Zeligowski A. (2014). Evolution in Four Dimensions: Genetic, Epigenetic, Behavioral, and Symbolic Variation in the History of Life. Cambridge, MA: The MIT Press. doi: 10.7551/mitpress/9689.001.0001

[B21] Jung C. G. (1959). The Archetypes and the Collective Unconscious. New York, NY: Pantheon Books. Available online at: http://archive.org/details/archetypescollec0009_part1 (Accessed April 16, 2026).

[B22] Kristeva J. (1982). Powers of Horror: An Essay on Abjection. New York, NY: Columbia University Press.

[B23] Meaney M. J. Szyf M. (2005). Environmental programming of stress responses through DNA methylation: life at the interface between a dynamic environment and a fixed genome. Dialogues Clin. Neurosci. 7, 103–123. doi: 10.31887/DCNS.2005.7.2/mmeaney 16262207 PMC3181727

[B24] Ongaro L. Huerta-Sanchez E. (2024). A history of multiple Denisovan introgression events in modern humans. Nat. Genet. 56, 2612–2622. doi: 10.1038/s41588-024-01960-y 39501127

[B25] Reich D. Green R. E. Kircher M. Krause J. Patterson N. Durand E. Y. . (2010). Genetic history of an archaic hominin group from Denisova Cave in Siberia. Nature 468, 1053–1060. doi: 10.1038/nature09710 21179161 PMC4306417

[B26] Roebroeks W. Villa P. (2011). On the earliest evidence for habitual use of fire in Europe. Proc. Nat. Acad. Sci. 108, 5209–5214. doi: 10.1073/pnas.1018116108 21402905 PMC3069174

[B27] Stringer C. (2012). The status of Homo heidelbergensis (Schoetensack 1908). Evol. Anthropol. Issues News Rev. 21, 101–107. doi: 10.1002/evan.21311 22718477

[B28] Stringer C. Gamble C. (1993). In Search of the Neanderthals: Solving the Puzzle of Human Origins. New York, NY: Thames and Hudson.

[B29] Trinkaus E. Shipman P. (1993). The Neandertals: Changing the Image of Mankind. London: Jonathan Cape.

[B30] Turner V. Abrahams R. Harris A. (1969). The Ritual Process: Structure and Anti-Structure. New York, NY: Routledge.

[B31] Vernot B. Akey J. M. (2014). Resurrecting surviving neandertal lineages from modern human genomes. Science 343, 1017–1021. doi: 10.1126/science.1245938 24476670

[B32] Yazigi J. B. Cyrino C. O. Peter C. M. Ferreira R. C. Maricato J. T. Janini L. M. . (2026). Oncogenic HPV types identified in Paleolithic and Chalcolithic human genome sequencing data from Ust'-Ishim and Ötzi. Sci Rep. 16:18045. doi: 10.1038/s41598-026-49280-x 42000811 PMC13254242

[B33] Yehuda R. Daskalakis N. P. Bierer L. M. Bader H. N. Klengel T. Holsboer F. . (2016). Holocaust exposure induced intergenerational effects on *FKBP5* methylation. Biol. Psych. 80, 372–380. doi: 10.1016/j.biopsych.2015.08.005 26410355

